# Psychometric Properties of the Coercion in Intimate Partner Relationships Scale

**DOI:** 10.1177/10731911211025628

**Published:** 2021-06-24

**Authors:** Kathleen D. Wilson, Patti A. Timmons Fritz

**Affiliations:** 1 University of Windsor, Windsor, Ontario, Canada

**Keywords:** intimate partner aggression, coercive control, validity, reliability, trauma symptoms

## Abstract

Coercive control is defined as the systematic use of demands, threats, and surveillance behaviors to gain control over an individual. Content validity appears to be an issue for existing measures of coercive control tactics, as they do not assess all of these behaviors. This study investigated the validity and reliability of the Demand, Threat, Surveillance, and Response to Demands subscales of the Coercion in Intimate Partner Relationships (CIPR) scale. Participants (*N* = 541) completed online measures of coercive control, physical intimate partner violence, depression, and posttraumatic stress disorder symptomatology. Confirmatory factor analyses, linear regressions, and correlational analyses investigated the construct (i.e., concurrent, convergent, and discriminant) validity of the CIPR subscales. Internal consistency of the subscales and test–retest reliability were also examined. Results provided support for the validity and reliability of the CIPR. Implications and usage of the CIPR in research and practice are discussed. We report how we determined our sample size, all data exclusions, all manipulations, and all measures in the study.

Intimate partner violence (IPV), defined as harmful physical, sexual, or psychological acts committed by a romantic partner, continues to be a global issue wherein women are typically victimized by men (World Health Organization, 2021). Approximately 30% of women worldwide have reported experiencing violence in the context of an intimate relationship, with victims commonly suffering physical injuries, psychological distress, and in some cases, death by suicide or homicide (World Health Organization, 2021). It is theorized that more severe types of IPV occur within the context of coercive control, herein referred to as coercion (Dutton & Goodman, 2005; Johnson, 1995).

Dutton and Goodman (2005) propose that coercion involves a demand made by a coercive partner (e.g., end a friendship), an associated threat or negative consequence for noncompliance (e.g., physical IPV), and surveillance of whether the partner has complied (e.g., checking text messages). The victim is hypothesized to respond to the coercion cognitively, emotionally, and behaviorally by either complying or not complying with the demand, which is influenced by the degree to which the target believes and fears the threat. Coercion is theorized to affect the victimized partners’ quality and outlook on life and physical and mental health.

Dutton and Goodman’s (2005) theory is consistent with French and Raven’s (1959) conceptualization of power in relationships and it was guided by a comprehensive literature review. It is also ecologically valid, as it was informed by ethnographic interviews with experts and individuals who have experienced IPV, a review of archival data (i.e., police reports and interviews with perpetrators of IPV), and consultation with experts in the field (Dutton et al., 2007).

The theorized relation between coercion and IPV has been supported in research with samples of divorced couples (Tanha et al., 2010), emerging adult men (Whitaker, 2013), high school students (Fawson, 2015), elderly persons (Policastro & Finn, 2017), and same-sex couples (Frankland & Brown, 2014). The relation between coercion and mental health, particularly depression and posttraumatic stress disorder (PTSD), has also been studied with various samples (Anderson, 2008; Bubriski-McKenzie & Jasinski, 2013; Coker et al., 2002; Johnson & Leone, 2005; Jones, 2020; Levine & Fritz, 2016; Lovestad et al., 2017; Terrazas-Carrillo et al., 2016).

Although the results of the extant literature are consistent with Dutton and Goodman’s (2005) theory, findings are limited in that the studies have not been guided by the same theoretical framework and coercion has not been measured using a standardized tool. In many cases, coercion has been measured using author-created items or broad measures of IPV (e.g., the Relationship Behavior Rating scale [Attala et al., 1994], the Restrictive subscale of the Dominance scale [Hamby, 1996], and the Revised Controlling Behaviors scale [Graham-Kevan & Archer, 2005]). Content validity appears to be an issue for the measures used in the research to date, as the measures do not assess demands, threats, and surveillance behaviors. This lack of standardization, as well as issues with making comparisons across studies, has recently been recognized by researchers in the field (Hamberger et al., 2017; Hardesty et al., 2015).

A potential solution to this problem is the standardized use of the Coercion in Intimate Partner Relationships scale (CIPR; Dutton et al., 2007). The CIPR is the only known instrument capable of measuring all of the tactics of coercion that have been theorized by Dutton and Goodman (2005). The CIPR is comprised of subscales that assess whether respondents have experienced or perpetrated demands, threats, and surveillance behaviors. It also assesses victim responses to demands and the involvement of other people. As such, the CIPR appears to have good content validity.

Other psychometric properties of the CIPR have been examined by Dutton et al. (2007) in a report for the National Institute for Justice (which has not undergone peer review). Dutton et al. (2007) used a sample of 750 men (*n* = 302) and women (*n* = 448) between the ages of 18 and 80 years (*M* age = 31 years) who had been in a romantic relationship for at least 1 year. Participants were either victims of IPV (*n =* 139), perpetrators of IPV (*n* = 39), both a victim and a perpetrator (*n* = 245), or had no personal experience with IPV (*n* = 334). Exploratory and confirmatory factor analyses supported the hypothesized factor structures of the Demand, Threat, Surveillance, and Response to Demand subscales of the CIPR. Support was also found for the construct validity of the CIPR and internal consistency was high, as Cronbach alphas were .86 or higher for all subscales.

The current study examined the psychometric properties (internal consistency; test–retest reliability; concurrent, convergent, and discriminant validity) of the CIPR using a community sample and an adjusted response scale. In response to research supporting the use of frequency-based scales to measure coercion (i.e., Hardesty et al., 2015), we changed the original *yes* or *no* scale to the 8-point Likert-type scale used in the well-known Revised Conflict Tactics Scales (Straus et al., 1996). Hypotheses were not made due to the exploratory nature of the study. However, based on Dutton and Goodman’s (2005) theory and Dutton et al.’s (2007) initial investigation of the CIPR we expected that the factor structure of the CIPR subscales (i.e., Demand, Threat, Surveillance, and Response to Demand) would replicate with the altered response scale when examined through confirmatory factor analyses. Although we anticipated that the chi-square goodness-of-fit statistics would be significant due to the large sample size, other fit indices were expected to indicate acceptable model fit. Model fit was assessed by the following indices and criteria: (a) root mean square error of approximation (RMSEA; ≤.05), (b) the standardized root mean square residual (SRMR; ≤.05), (c) the nonnormed fit index/Tucker–Lewis index (NNFI/TLI; ≥.90), and (d) the comparative fit index (CFI; ≥.90).

Furthermore, we anticipated that higher coercion scores on the CIPR would be associated with higher scores on the Checklist of Controlling Behaviors (CCB; Lehmann et al., 2012), demonstrating concurrent validity (Dutton et al., 2007). In terms of convergent validity, we expected that higher scores on overall coercion, as measured by the CIPR, would be associated with higher scores on physical IPV, as measured by the CCB (Fawson, 2015; Tanha et al., 2010). We expected that higher total coercion victimization would be associated with higher depression and PTSD symptomatology scores, as was found in the studies described above. We also anticipated that scores on the Demand, Threat, and Surveillance subscales would be positively related (Dutton et al., 2007). Moreover, we expected that scores on a measure of socially desirable answering would not be correlated with coercion to an extent that would suggest that the instruments assess the same construct (i.e., *r* ≥ .50; Pituch & Stevens, 2016), demonstrating discriminant validity.

In terms of reliability, we expected that the CIPR would demonstrate high internal consistency, with Cronbach alpha scores at or above .70 for all subscales (Dutton et al., 2007; George & Mallery, 2005). Finally, we expected that good test–retest reliability would be apparent as demonstrated via coefficients that are at or above .60 (Cicchetti & Sparrow, 1981).

## Method

### Participants

Participants consisted of 625 self-identified men (41%) and women (59%) between the ages of 18 and 71 (*M* =35.22, *SD* = 11.89) years. Participants were recruited online via a pool of university students who received course credit in eligible courses in exchange for participating in research at a university in Southwestern Ontario, Canada (*n* = 76) as well as through Mechanical Turk (MTurk; *n* = 549). Inclusion criteria necessitated that participants resided in Canada or the United States, had been in a romantic relationship for at least 3 months, and were not in long-distance or online relationships. MTurk workers and university students identified as Caucasian (74% and 80%, respectively), African American/Black Canadian (15% and 4%, respectively), Hispanic/Latinx (6% and 1%, respectively), and East Asian/Pacific Islander (5% and 10%, respectively). MTurk workers and university students identified as heterosexual (84% and 87%, respectively), bi-sexual (12% and 9%, respectively), and lesbian/gay (3% and 1%, respectively).

In order to examine if participants recruited through MTurk and those recruited through the university pool differed on important demographics and study variables, we ran a series of independent *t* tests, Mann Whitney *U* tests, and chi-square tests. The two groups did not differ on the majority of study variables; however, the proportion of Black participants to non-Black participants was lower in the MTurk group (5:1) as compared with the university sample (22:1). MTurk participants reported significantly more coercion and violence perpetration as compared with university students and MTurk participants were also more likely than university students to believe their partners’ threats and be more fearful in response to threats made by their partners.

Differences were also found for age wherein MTurk participants were significantly older (*M* age = 36.94) than university participants (*M* age = 23.47). Moreover, a greater proportion of MTurk workers had graduated from post-secondary education as compared to university students. Last, gender was more evenly distributed in the MTurk sample (1.0:1.2) as compared with the university sample (1.0:9.0), which consisted primarily of women. Overall, recruiting university students and MTurk workers was successful in providing a more diverse sample. Descriptive statistics for the final, combined sample can be referenced in Table 1.

**Table 1.** Means, Medians, Standard Deviations, and Cronbach’s Alpha Values for Main Study Variables.

Variable	Mean/Median (*SD*)	Cronbach’s alpha
PTSD	43.10 (19.74)	.97
Depression	14.52 (5.05)	.88
Socially Desirable Answering	6.16 (3.16)	.75
CCB total coercion victimization	83.00 (76.32)^a^	.99
CCB total coercion perpetration	82.00 (66.32)^a^	.99
CIPR total coercion victimization	128.00 (151.11)^a^	.86
CIPR total coercion perpetration	110.00 (143.15)^a^	.86
CIPR demand victimization	79.00 (70.47)^a^	.98
CIPR surveillance victimization	18.00 (24.14)^a^	.96
CIPR threat victimization	31.00 (51.16)^a^	.99
CIPR response to demand	25.00 (25.95)^a^	.96
CIPR demand perpetration	63.00 (73.19)^a^	.98
CIPR surveillance perpetration	14.00 (24.20)^a^	.97
CIPR threat perpetration	31.00 (54.00)^a^	.99

*Note*. PTSD = posttraumatic stress disorder; CCB = Checklist of Controlling Behaviors; CIPR = Coercion in Intimate Partner Relationships scale.

a Denotes median.

### Measures

#### Coercion

The 202-item CIPR (Dutton et al., 2007) scale was used to examine victimization and perpetration of coercion in the context of one’s current romantic relationship. Demands, threats, and surveillance tactics used in the last 3 months of the relationship were assessed. Specifically, the 48-item Demand subscale assessed demands made regarding personal activities and appearance (e.g., maintaining a certain weight); support and social life (e.g., spending time with friends or family members); household activities (e.g., taking care of the house); work, economics, and resources (e.g., going to school); health (e.g., taking medication or prescription drugs); the intimate relationship (e.g., doing certain sexual behaviors); legal matters (e.g., talking to the police or lawyer); immigration (e.g., talking to the immigration authorities); and children (e.g., making important decisions about the children). The 31-item Threat subscale assessed threats that fit into the categories of (a) harm to the participant (e.g., physically hurt you), (b) harm to the perpetrator/partner (e.g., threaten to commit suicide), and (c) harm to others (e.g., destroy property of family members or friends). The 13-item Surveillance subscale assessed a variety of means of checking on someone’s activities (e.g., kept track of telephone/cell phone use; checked victim’s clothing). Finally, the 16-item Response to Demand subscale assessed participants’ reactions to their partners’ demands (e.g., argued back).

With permission from the author, (M. A. Dutton, personal communication, April 30, 2018), the response scale of the CIPR was altered to the following: 1 (*This has never happened*), 2 (*Not in the past 3 months, but it did happen before*), 3 (*Once in the past 3 months*), 4 (*Twice in the past 3 months*), 5 (*3-5 times in the past 3 months*), 6 (*6-10 times in the past 3 months*), 7 (*11-20 times in the past 3 months*), and 8 (*More than 20 times in the past 3 months*). The instructions provided before each subscale were also altered slightly to reflect the change in obtaining information about frequency. Subscale scores were derived for the Demand, Threat, Surveillance, and Response to Demand subscales by summing all items within each subscale. We also calculated total coercion perpetration and total victimization scores, which were the sums of all subscale scores. Higher scores indicated a greater amount of coercion. Initial psychometric testing of the CIPR has demonstrated high reliability (α ≥ .86) and validity (Dutton et al., 2007).

#### Other Components and Information About Coercion

Participants were asked to report on the extent to which they believed that their partner would follow through with threatened negative consequences for not complying with demands, fear related to their partners’ coercive behaviors, the frequency with which they experienced and perpetrated coercion, and estimates of when coercive behaviors were first observed in current relationships. This information was collected to inform future research and will not be reported.

#### Controlling Behaviors and Intimate Partner Violence

The CCB (Lehmann et al., 2012) was used to assess victimization and perpetration of control tactics, as well as physical and psychological IPV. Using a 5-point Likert-type scale ranging from 0 (*never*) to 4 (*very frequently*), participants were asked to indicate how often, over the past three months of their current relationship, they were the victims and perpetrators of various forms of physical (10 items; e.g., choked me), sexual (9 items; e.g., physically forced me to have sexual intercourse), emotional (9 items; e.g., told me I was crazy), and economic (7 items; e.g., made me ask for money for the basic necessities) abuse, as well as how often control tactics were used, including threats (7 items, e.g., to hit or kill me), intimidation (7 items; e.g., smashed or broke something), minimizing of abuse (7 items; e.g., told me that abuse was a normal part of relationships), blaming (7 items; e.g., blamed me for his or her abusive behavior saying it was my fault), isolation (10 items; e.g., forbade me or stopped me from seeing someone), and male privilege or inferiority (9 items; e.g., treated me like a servant). Instructions were altered slightly to reflect that not all participants in the current study were in abusive relationships. Additionally, in order to make the items clearer, we added “my partner*”* to the beginning of the victimization items and “I” to the beginning of the perpetration items. We calculated a total sum score across all items separately for victimization and perpetration and higher scores indicated a greater amount of coercion. Previous research indicates that the CCB is a reliable (α ≥ .80) and valid tool (Lehmann et al., 2012).

#### Depression

The Quick Inventory of Depressive Symptomology–Self-Report (QIDS-SR; Rush et al., 2003) was used to assess the existence of current symptoms of depression, including sleep, mood, appetite, weight, concentration, energy, and interests. An item about thoughts of death or suicide was not included in the current study for ethical reasons. Participants were asked to choose the answer that best described their state over the past 1 or 2 weeks, depending on the item. Participants answered using a 4-point Likert-type scale that varied from item to item. A total score was derived by summing the items according to Rush et al.’s (2003) guidelines. Higher scores indicated more severe depression. The QIDS-SR has been found to be a reliable (α= .86) and valid instrument (Rush et al., 2003; Trivedi et al., 2004).

#### Posttraumatic Stress Disorder

The PTSD Checklist for *DSM-5* (PCL-5; Weathers et al., 2013) was used to assess the existence of current *Diagnostic and Statistical Manual of Mental Disorders–Fifth edition* (*DSM-5*; American Psychiatric Association, 2013) symptoms of PTSD, including: intrusive memories and dreams, avoidance behaviors, physiological reactions to cues of the stressful event, alertness, blame, and negative emotions. Participants rated how much they had been bothered by each issue over the past month using a 4-point Likert-type scale that ranged from 0 (*not at all*) to 4 (*extremely*). We calculated a total sum score and higher scores indicated more symptoms of PTSD. The PCL-5 has been found to be a reliable (α= .94) and valid tool (Blevins et al., 2015).

#### Demographics

Among other demographics, participants were asked questions regarding their ethnicity, sexuality, dating history, and past experiences of IPV. Participants were also asked to report on their current relationship, including the length of the relationship, relationship satisfaction, and their relationship/cohabitation status.

#### Socially Desirable Responding

The 13-item Marlowe–Crowne Social Desirability Scale Short-Form C (MCSDS Form C; Reynolds, 1982) was used to assess participants’ tendencies to answer questions in a socially desirable manner. Participants rated whether statements regarding personal attitudes and traits were *true* (0) or *false* (1) of them. We calculated a total sum score after reverse coding five items. The MCSDS Form C has been found to be a reliable (α= .67) and valid instrument (Reynolds, 1982).

#### Validity Checks

In order to determine if participants were dedicating their full attention toward the task, four validity check questions were added throughout the survey (e.g., please choose response “never” for this question). If participants did not choose the answer asked of them for the majority of the validity checks (i.e., >50%), their data were excluded (*n* = 8).

### Procedure

#### University Recruitment

Once clearance was obtained from the university’s Research Ethics Board, we posted online study advertisements to the university participant pool website. In order to collect data to assess test–retest reliability of the CIPR, the study was advertised as a two-part study. For financial reasons, test–retest reliability was only tested with the university sample. Part 1 of the study took approximately 35 minutes to complete and participants were awarded with one bonus credit, which was added to their mark in an eligible class. Students earned an additional 0.50 of a credit for completing only the CIPR again (Part 2), which took an average of 10 minutes and was completed 2 weeks after Part 1.

#### Mechanical Turk Recruitment

We also posted online study advertisements on the MTurk website and interested MTurk workers who met the inclusion criteria signed up through their worker accounts. MTurk workers earned a one-time stipend of US$1.25 for completing Part 1 of the study, which took an average of 35 minutes to complete.

#### Data Collection

After MTurk and university participants provided informed consent, they completed the questionnaires online through Qualtrics. Questionnaires were presented in randomized order to control for order effects. On completion of the questionnaires, participants completed a positive mood induction procedure wherein they wrote about a positive memory that they had of their current partner or romantic relationship (Trope et al., 2001). These responses were not coded and are not included in the current study. Following this, participants were directed to a list of IPV and mental health resources, as well as instructions for how to clear one’s internet browser history for safety purposes.

## Results

### Preliminary Analyses

Before data were collected, we specified and identified each of the following models (i.e., CIPR subscales) and determined whether a unique solution was possible for each. The Demand (perpetration and victimization) models consisted of nine factors/exogenous latent variables (i.e., Personal Activities and Appearance, Support and Social Life, Household, Work/Economics and Resources, Health, Intimate Relationship, Legal, Immigration, and Children/Parenting) and 48 items/endogenous variables. Each of the nine latent factors was theorized to have a different number of items. The Threat (perpetration and victimization) models consisted of three factors/exogenous latent variables (i.e., Harm to you, Harm to Partner, and Harm to Others) and 31 items/endogenous variables. Each of the three latent factors was theorized to have a different number of items. The Surveillance (perpetration and victimization) models consisted of one factor/exogenous latent variable and 13 items/endogenous variables. The Response to Demand (victimization only) model consisted of two unnamed factors/exogenous latent variables and 16 items/endogenous variables. Each of the two latent factors was theorized to have a different number of items. Subscale items and factor loadings can be referenced in Supplemental Tables S1 to S4 (available online).

The models of the CIPR were assumed to be properly specified, as the CIPR is grounded in theory, research findings, and ethnographic interviews. Moreover, exploratory and confirmatory factor analyses were initially conducted by the authors of the CIPR. The difference between the number of observed variables in each of the models (p*) and the number of parameters that needed to be estimated was calculated for each model and each of the models were found to be over identified (Pituch & Stevens, 2016).

Once the data set was cleaned (*N* = 548; *n* for MTurk = 479; *n* for university pool = 69), missing data and assumptions were examined. Little’s MCAR test indicated that data were not missing completely at random, χ^2^(189) = 278.21, *p* < .001. The most frequent patterns of missing data were no missing data, followed by missing CIPR perpetration scores and the combination of missing CIPR perpetration scores and CIPR victimization scores. The second and third patterns affected less than 10% of cases. To determine whether the missing data were missing at random, we created dichotomous missing data variables for CIPR total victimization and CIPR total perpetration. We examined whether participants who did and did not have these scores differed significantly on any of the other main variables of interest or demographic variables. Results of a series of *t* tests, Mann Whitney *U* tests, and chi-square tests indicated that participants who did and did not have CIPR total victimization and perpetration scores differed significantly on study variables and some of the demographics (e.g., education level), but not on the other measure of coercion (i.e., the CCB). Therefore, data appeared to be missing at random, as a systematic relationship existed between the propensity of missing values and other observed data, but not to coercion itself. We also examined item-level missing data for the CIPR items. All CIPR items were missing less than 2% of data, or five cases. Listwise deletion was used for the confirmatory factor analyses because CIPR missing data were minimal at the item-level (Pituch & Stevens, 2016). To obtain a full data set for the correlational and linear regression analyses, we used multiple imputation with 10 imputations.

Next, data were assessed for multivariate outliers using a Mahalanobis Distance Test (Tabachnick & Fidell, 2013). Seven multivariate outliers were identified and removed (*N* = 541; *n* for MTurk = 472; *n* for university pool = 69). Assumptions of linear regression were examined, including: normality (accomplished via visual analysis of plot of residuals and examination of values of skewness and kurtosis), homoscedasticity (accomplished via visual analysis of plot of standardized residuals and standardized predicted values), and linearity (accomplished via visual analysis of scatterplot). All assumptions were met and no univariate or influential outliers were identified. Prior to running the confirmatory factor analyses, we assessed normality of the CIPR items (accomplished via the Kolmogorov-Smirnov test and an examination of values of skewness and kurtosis). Normality was found to be violated for all CIPR items. In response to this, a Satorra–Bentler correction was used when conducting the confirmatory factor analyses.

### Primary Analyses

We examined the factor structures of the CIPR subscales through confirmatory factor analyses separately for perpetration and victimization (using SAS university edition). Within these domains, analyses were performed for the Demand, Threat, and Surveillance subscales. We also conducted an analysis for the Response to Demand subscale (victimization only). Covariance matrices and the maximum likelihood function were used when conducting the analyses and the unit variance identification method was used for all models, with variances directly set to a value of one. Error was also scaled by fixing the direct paths to their corresponding endogenous variables to one (Pituch & Stevens, 2016). The latent variables were allowed to co-vary. The results of these analyses can be found in Table 2. Although the chi-square-goodness-of-fit-statistics were significant, likely due to an abundance of statistical power, other fit statistics indicated good model fit for all subscales. Moreover, the inclusion of RMSEA and NNFI/TLI indices, which adjust for model complexity (Pituch & Stevens, 2016), countered the potential problem of better fit for models that required more estimation. Taken together, it appears as though the items within each of the CIPR subscales are valid for measuring demands (and reactions to demands), threats, and surveillance behaviors within romantic relationships. Notably, these results and those outlined below were consistently found when the data set was randomly split into two samples.

**Table 2.** Fit Indices for the CIPR Subscales.

Fit indices						
CIPR subscale	SB chi-square	RMSEA	SRMR	NNFI/TLI	CFI	Overall fit
Demand victimization	χ^2^(1044) = 3999.15, *p* < .001	0.04, 90% CI [0.03, 0.04]	0.05	0.93	0.93	Good fit
Threat victimization	χ^2^(431) = 4427.49, *p* < .001	0.04, 90% CI [0.04, 0.05]	0.03	0.95	0.95	Good fit
Surveillance victimization	χ^2^(65) = 592.43, *p* < .001	0.06, 90% CI [0.05, 0.07]	0.04	0.96	0.97	Good fit
Victim response to demand	χ^2^(103) = 1006.85, *p* < .001	0.07, 90% CI [0.06, 0.07]	0.06	0.93	0.94	Good fit
Demand perpetration	χ^2^(1044) = 4310.68, *p* < .001	0.03, 90% CI [0.03, 0.04]	0.04	0.93	0.95	Good fit
Threat perpetration	χ^2^(431) = 4437.44, *p* < .001	0.03, 90% CI [0.03, 0.04]	0.02	0.97	0.97	Good fit
Surveillance perpetration	χ^2^(65) = 620.00, *p* < .001	0.06, 90% CI [0.05, 0.07]	0.03	0.96	0.97	Good fit

*Note.* SB chi-square nonsignificance indicates good fit. RMSEA ≤ .05 indicates good fit; .05-.08 indicates acceptable fit. SRMR ≤ .05 indicates good fit; .05-.10 indicates acceptable fit. NNFI/TLI ≥ .90 indicates good fit. CFI ≥ .90 indicates good fit. SB chi-square = Satorra–Bentler chi-square goodness-of-fit index; RMSEA = root mean square error of approximation; SRMR= standardized root mean-square residual; NNFI/TLI = non-normed fit index/Tucker Lewis index; CFI = comparative fit index; CI = confidence interval.

To assess the concurrent validity of the CIPR, we conducted Spearman’s Rank correlational analyses with the CCB, which measures control tactics. Specifically, total coercion perpetration as measured by the CCB was correlated with total coercion perpetration as measured by the CIPR (ρ = .71, *p* ≤ .001), demand perpetration (ρ = .71, *p* ≤ .001), threat perpetration (ρ = .75, *p* ≤ .001), and surveillance perpetration (ρ = .74, *p* ≤ .001). Total coercion victimization as measured by the CCB was also correlated with coercion victimization as measured by the CIPR (ρ = 0.68, *p* ≤ .001), demand victimization (ρ = 0.64, *p* ≤ .001), threat victimization (ρ = 0.76, *p* ≤ .001), and surveillance victimization (ρ = 0.67, *p* ≤ .001). As expected, higher coercion perpetration and victimization subscale and composite scores on the CIPR were significantly related to higher coercion perpetration and victimization scores on the CCB. These analyses and those described below were completed using SPSS version 22.0.

In order to assess the convergent validity of the CIPR, Spearman’s Rank correlational analyses were conducted using the physical IPV subscale of the CCB. Specifically, physical IPV perpetration as measured by the CCB was correlated with total coercion perpetration as measured by the CIPR (ρ = .70, *p* ≤ .001), demand perpetration (ρ = .69, *p* ≤ .001), threat perpetration (ρ = .77, *p* ≤ .001), and surveillance perpetration (ρ = .73, *p* ≤ .001). Physical IPV victimization as measured by the CCB was also correlated with coercion victimization as measured by the CIPR (ρ = .71, *p* ≤ .001), demand victimization (ρ = .66, *p* ≤ .001), threat victimization (ρ = .76, *p* ≤ .001), and surveillance victimization (ρ = .70, *p* ≤ .001). As expected, results indicated that higher coercion perpetration and victimization subscale and composite scores on the CIPR were significantly related to higher physical IPV perpetration and victimization scores on the CCB.

To further assess convergent validity, we conducted linear regression analyses between CIPR coercion victimization and PTSD as well as between CIPR victimization and depression. As expected, coercion victimization was related to PTSD (β = .65; *b* = .09, 95% confidence interval [0.08, 0.10], *p* ≤ .001) and it accounted for 42% of the variance in PTSD symptomatology, *F*(1, 540) = 383.97, *p* ≤ .001. Coercion victimization was also related to depression (β = .44; *b* = .02, 95% confidence interval [0.01, 0.02], *p* ≤ .001) and it accounted for 20% of the variance in depression symptomatology, *F*(1, 540) = 131.55, *p* ≤ .001. As expected, coercion victimization over the past 3 months was related to current PTSD and depression symptomatology.

Finally, Spearman’s rank correlational analyses examined the relation between the Demand, Threat, and Surveillance subscales. All CIPR subscales were significantly positively related to one another at an alpha level of .01. This was true within and across perpetration and victimization. Moreover, all correlations were at or above ρ = .67, with the majority being above ρ = .70 (see Supplemental Table S5, available online).

In order to assess the discriminant validity of the CIPR, we ran Spearman’s rank correlations with the measure of socially desirable responding (i.e., the MCSDS). Specifically, total MCSDS scores were correlated with total coercion perpetration (ρ = −.13, *p* = .05) and victimization (ρ = −.12, *p* = .05) as measured by the CIPR. As expected, total CIPR victimization and total perpetration scores were not correlated with total MCSDS scores to an extent that would suggest singularity or that the instruments were measuring the same construct (i.e., ρs ≥ 0.50; Pituch & Stevens, 2016).

In order to examine the internal consistency of the CIPR subscales, we derived Cronbach’s alpha statistics. As can be referenced in Table 1, results indicated that the CIPR subscales have either good or excellent internal consistency (George & Mallery, 2005).

We also examined 2-week test–retest reliability by conducting Spearman’s rank correlations using total CIPR coercion perpetration and victimization scores from university students’ initial survey response and their total CIPR coercion perpetration and victimization score from their second survey response two weeks later. Responses from a total of 42 participants were included in the analyses. A series of *t* tests and Mann Whitney *U* tests were conducted to examine whether university students who completed the 2-week retest portion of the study differed from those who did not complete the retest portion on main study variables. Participants did not differ on age or on any of the measures of interest. Test–retest reliability for coercion perpetration was excellent, ρ = 0.79, *p* ≤ .001 and test–retest reliability for coercion victimization was good, ρ = 0.62, *p* ≤ .001 (Cicchetti & Sparrow, 1981).

## Discussion

The current study provides support for the validity of Dutton and Goodman’s (2005) theory of coercion, as good model fit was found for the subscales of the CIPR and total CIPR coercion victimization was related to mental health outcomes. Consistent with Dutton et al.’s (2007) initial examination of the CIPR, the current study also provides further empirical evidence for the measure’s construct validity and reliability. Specifically, confirmatory factor analyses supported the factor structure of the CIPR subscales with the altered response scale. As expected, the CIPR coercion victimization and perpetration subscales were related to one another, another measure of coercion, and physical violence, but not to a measure of socially desirable answering. Coercion victimization was also related to depression and PTSD symptomatology. As such, evidence was found for the concurrent, convergent, and discriminant validity of the CIRP subscales. Internal consistency for all of the CIPR subscales ranged from good to excellent and test–retest reliability was good for coercion victimization and excellent for coercion perpetration. Not surprisingly, this finding indicates that the participants who completed the two administrations were more reliable at reporting their own behaviors as compared to their partners’ behaviors (Armstrong et al., 2002).

Taken together, results support the use of the frequency-based CIPR by researchers studying coercion. Not only is the CIPR a psychometrically defensible tool but it is also the only known instrument designed to measure all of the components of coercion proposed by Dutton and Goodman (2005). Thus, if the tool is adopted by researchers, knowledge of coercion and its correlates will likely be improved as the CIPR will better capture the complexity of coercion.

### Limitations and Future Directions

The current study was not without limitations. Although support was found for the validity and reliability of the CIPR, the sample used was composed primarily of participants recruited from the United States, where the measure was created. Findings may not be generalizable to people living in other countries. For instance, demands, threats, or surveillance tactics that are used by romantic partners from the United States may not be the same as those used in other areas of the world. Moreover, the sample used to examine test–retest reliability was small and composed primarily of women enrolled in a university in Southwestern Ontario, Canada. As such, the findings related to test–retest reliability may not be generalizable and should be interpreted with caution, as a larger sample is needed to properly determine test–retest reliability. Future studies should examine the validity and reliability of the CIPR using larger and more diverse samples. Examinations using clinical and forensic samples would be particularly important in order to determine whether the CIPR would be useful in clinical or criminal justice settings.

Furthermore, in the current study participants were asked to report on their partners’ behaviors. This reporting method likely introduced error, as participant answers could have been affected by lapses in memory or biases in the way they viewed situations with their partner. Furthermore, test–retest reliability results suggested that participants were more reliable at reporting their own behaviors, as compared with the behaviors of their partners. To remedy this, both partners in a romantic relationship should be recruited in future investigations.

Another important future direction is shortening the CIPR to a length that will be more appropriate for research, especially much-needed longitudinal work. Once a shortened version has been created, clinical severity of coercion could be measured in a theoretically driven way by adding questions that address threat appraisal and fear response. Furthermore, as suggested by Hardesty et al. (2015), standardized cut-off values distinguishing between high versus low coercion would be advantageous.

### Possible Implications

If the CIPR is found to be a valid and reliable tool for use with clinical and forensic populations, there may be important uses for the tool outside of research. For instance, the current finding that coercion victimization is related to PTSD and depression symptomatology suggests that individuals who have been in an abusive relationship characterized by coercion may be at-risk for experiencing psychological distress or vice versa. As such, these individuals may benefit from talking to a mental health, advocacy, or community support provider. Special caution should be taken not to interpret these findings as a means for labeling or pathologizing victims’/survivors’ responses to traumatic experiences, but to instead highlight that psychological distress is a normal and common reaction to coercion and that services and supports are available to survivors who may want to seek them out. Thus, if the CIPR is found to be a valid and reliable tool in clinical samples, service providers could use the CIPR to inquire about whether clients in abusive relationships have been bothered by demands, threats, and surveillance behaviors made by romantic partners. Moreover, clinicians working with psychologically distressed clients could also use the CIPR to inquire about IPV and coercion. Information such as this could enhance the care providers’ conceptualization of the situation and inform treatment, advocacy, and service provision.

If found to be a valid and reliable tool in forensic samples, the CIPR could potentially be used to identify perpetrators who would benefit from specialized correctional treatment programs. Day and Bowen (2015) propose that perpetrators who use IPV in the context of coercion hold qualitatively different beliefs about violence as compared with perpetrators of noncoercive IPV. Moreover, they propose that the origin of coercive violence is different than that of noncoercive violence and that perpetrators of coercion are characterized by traits of psychopathy, making it more difficult to change the beliefs and behaviors of perpetrators of coercion as compared with perpetrators of noncoercive IPV. In an attempt to move away from a one-size-fits-all treatment approach and toward more effective interventions, Day and Bowen (2015) propose that specialized interventions be created for perpetrators of coercion. Once these interventions have been created, the CIPR might be an appropriate screening tool to determine whether perpetrators are coercive as well as which program would be most instrumental for behavioral change based on the type of perpetrator.

In terms of a potential preventative implications of the current study, Dutton and Goodman’s (2005) theory could be shared with adolescents to educate them about unhealthy patterns of behaviors in romantic relationships. Sargent et al. (2017) recently examined the effectiveness of TakeCare, which is a high school-based bystander intervention program targeting teen dating relationship violence (i.e., jealousy and control, heated arguments, sexual assault, physical violence, insults). The video-based program was effective at increasing positive bystander behaviors. However, although jealousy and control were the most prevalent acts of relationship violence at baseline and follow-up (44% and 38%, respectively), the intervention did not include modules and vignettes that covered control. Future renditions of this program or similar programs would likely be enhanced by adding modules that cover coercion as theorized by Dutton and Goodman (2005). Through education, it is possible that in the future, young adults might be more capable of identifying whether they or their partners are being controlling and act to change these behaviors or leave unhealthy situations.

## Conclusion

The current study examined the construct validity of the CIPR as well as the internal consistency and test–retest reliability of the instrument. This study was the first to examine the psychometric properties of the CIPR with a frequency-based response scale. The results of a series of confirmatory factor analyses, correlational analyses, and linear regression analyses provide support for the validity and reliability of the CIPR. Although this study was not without limitations, the findings support the use of the CIPR as a valid and reliable way to measure coercion in a theory-driven and standardized way.

## Supplemental Material

sj-pdf-1-asm-10.1177_10731911211025628 – Supplemental material for Psychometric Properties of the Coercion in Intimate Partner Relationships Scale Click here for additional data file. Supplemental material, sj-pdf-1-asm-10.1177_10731911211025628 for Psychometric Properties of the Coercion in Intimate Partner Relationships Scale by Kathleen D. Wilson and Patti A. Timmons Fritz in Assessment
