# Supplementary material for: Psychometric Properties of the Coercion in Intimate Partner Relationships Scale
Source: Assessment. 2021 Jun 24;30(2):448–57. doi: 10.1177/10731911211025628 (PMC9900190; doi:10.1177/10731911211025628)
Supplement: sj-pdf-1-asm-10.1177_10731911211025628 – Supplemental material for Psychometric Properties of the Coercion in Intimate Partner Relationships Scale [file sj-pdf-1-asm-10.1177_10731911211025628.pdf]

Table S1

Items and Standardized Factor Loadings for the Demand Subscale of the Coercion in Intimate Partner Relationships Scale (Victimization/Perpetration)

**Factor 1:** Personal Activities & Appearance	**Factor 2:** Support, Social Life, & Family	**Factor 3:** Household	**Factor 4:** Work, Economics, & Resources	**Factor 5:** Health	**Factor 6:** Intimate Relationship	**Factor 7:** Legal	**Factor 8:** Immigration	**Factor 9:** Children & Parenting
1. Leaving the house (.71/.77)	11. Talking on the phone (.80/.79)	17. Taking care of the house (.65/.75)	20. Working (.76/.81)	25. Using street drugs (.86/.84)	29. Talking to your partner (.61/.67)	37. Talking to police or lawyer (.86/.88)	41. Filing citizenship papers (.90/.87)	44. Taking care of children (.85/.83)
2. Eating (.75/.76)	12. Spending time with friends/family (.72/.77)	18. Buying or preparing foods (.62/.73)	21. Spending money, using credit cards, or bank accounts (.64/.74)	26. Using alcohol (.78/.81)	30. Spending time with your partner (.62/.64)	38. Doing things that are against the law (.88/.87)	42. Talking to the immigration authorities (.89/.91)	45. Disciplining the children (.88/.85)
3. Sleeping in certain places/times (.77/.82)	13. Going to church, school, or other community activities (.77/.80)	19. Living in certain places (.82/.88)	22. Learning another language (.80/.87)	27. Going to the doctor (.75/.80)	31. Separating or leaving the relationship (.82/.86)	39. Carrying a gun or knife (.86/.86)	43. Immigration sponsorship (.88/.89)	46. Making every day decisions about the children (.85/.89)
4. Wearing certain clothes (.79/.81)	14. Talking to a counselor, clergy, or someone else about personal or family matters (.81/.85)	--	23. Going to school (.75/.84)	28. Taking medication or prescriptions drugs (.76/.85)	32. Having sex (.68/.72)	40. Talking to landlord or housing authorities (.86/.89)	--	47. Making important decisions about the children (.87/.89)

**Factor 1:** Personal Activities & Appearance	**Factor 2:** Support, Social Life, & Family	**Factor 3:** Household	**Factor 4:** Work, Economics, & Resources	**Factor 5:** Health	**Factor 6:** Intimate Relationship	**Factor 7:** Legal	**Factor 8:** Immigration	**Factor 9:** Children & Parenting
5. Maintaining a certain weight (.85/.81)	15. Taking care of dependent relatives (.80/.87)	--	24. Using the car or truck (.81/.86)	--	33. Using birth control/condoms (.77/.74)	--	--	48. Talking to child protection authorities (.81/.83)
6. Using TV, radio, or the internet (.72/.79)	16. Taking care of pets (.73/.79)	--	--	--	34. Doing certain sexual behaviors (.69/.79)	--	--	--
7. Viewing sexually explicit material (.78/.85)	--	--	--	--	35. Having sex in exchange for money, drugs, or other things (.84/.87)	--	--	--
8. Bathing or using the bathroom (.81/.82)	--	--	--	--	36. Photographing you nude or while having sex (.83/.84)	--	--	--
9. Answering the phone (.74/.81)	--	--	--	--	--	--	--	--
10. Reading certain things (.81/.84)	--	--	--	--	--	--	--	--

Note. Factor loadings are presented in parentheses, with loadings for victimization on the left and loadings for perpetration on the right.

Table S2

Items and Standardized Factor Loadings for the Threat Subscale of the Coercion in Intimate Partner Relationships Scale (Victimization/ Perpetration)

**Factor 1:** Harm to You	**Factor 2:** Harm to Self/Partner	**Factor 3:** Harm to Others
1. Say something mean, embarrassing or humiliating to you (.72/.81)	26. Threaten to commit suicide (.90/.90)	28. Say something mean or hurtful to your friends or family members (.92/.89)
2. Keep you from seeing or talking to family or friends (.88/.91)	27. Actually attempt to harm or kill himself/herself (.89/.92)	29. Physically hurt a friend or family member (.91/.93)
3. Tell someone else personal or private information about you (.86/.91)	--	30. Try to kill a friend or family member (.91/.89)
4. Keep you from leaving the house (.84/.88)	--	31. Destroy property of family members or friends (.92/.93)
5. Limit your access to transportation (.88/.94)	--	--
6. Physically hurt you (.90/.91)	--	--
7. Try to kill you (.88/.90)	--	--
8. Scare you (.86/.89)	--	--
9. Have sex with someone else (.89/.91)	--	--
10. Leave the relationship or get a divorce (.90/.89)	--	--
11. Not let you take medication (.92/.93)	--	--
12. Put you in a mental hospital (.92/.94)	--	--
13. Cause you to lose your job (.90/.93)	--	--
14. Keep you from going to work (.87/.94)	--	--
15. Cause you to lose your housing (.90/.93)	--	--
16. Hurt you financially (.88/.93)	--	--
17. Cause you legal trouble (.92/.95)	--	--
18. Have you arrested (.90/.91)	--	--
19. Threaten to have you deported (.93/.92)	--	--
20. Force you to engage in unwanted sex acts (.72/.93)	--	--

**Factor 1:** Harm to You	**Factor 2:** Harm to Self/Partner	**Factor 3:** Harm to Others
21. Force you to participate in or observe sex acts with others (.91/.93)	--	--
22. Destroy legal papers (.90/.91)	--	--
23. Destroy or take something that belongs to you (.88/.92)	--	--
24. Physically hurt or kill your pet or other animal (.91/.92)	--	--
25. Not let you see your child or take your children from you (.92/.92)	--	--

Note. Factor loadings are presented in parentheses, with loadings for victimization on the left and loadings for perpetration on the right.

Table S3

Items and Standardized Factor Loadings for the Surveillance Subscale of the Coercion in Intimate Partner Relationships Scale (Victimization/Perpetration)

**Factor 1:** Surveillance
1. Checked or opened your mail or personal papers/journal (.85/.83)
2. Kept track of telephone/cell phone use (.86/.87)
3. Called you on the phone (.48/.56)
4. Told you to carry a cell phone or pager (.83/.85)
5. Checked your clothing (.85/.90)
6. Checked the house (.83/.88)
7. Checked receipts/checkbook/bank statements (.83/.83)
8. Checked the car (odometer, where parked) (.88/.93)
9. Asked the children, neighbors, friends, family or coworkers (.88/.91)
10. Told you to report your behavior to him/her (.90/.91)
11. Used audio or video tape recorder (.86/.89)
12. Spied on, followed, or stalked you (.87/.91)
13. Your partner didn't need to check; your partner just acted like he/she knew (.80/.81)

Note. Factor loadings are presented in parentheses, with loadings for victimization on the left and loadings for perpetration on the right.

Table S4

Items and Standardized Factor Loadings for the Victim Response to Demands Subscale of the Coercion in Intimate Partner Relationships Scale

**Factor 1: Unnamed**	**Factor 2: Unnamed**
1. Did what your partner wanted, even though you didn't want to (.63)	8. Fought back physically (.93)
2. Refused to do what he/she said (.75)	9. Used/threatened to use a weapon against him/her (.91)
3. Tried to talk your partner out of wanting you to do it (.77)	14. Filed for a civil protection order (.92)
4. Lied about having done what your partner wanted (.87)	15. Called the police (.92)
5. Sought help from someone else (.87)	16. Tried to get criminal charges filed (.93)
6. Tried to distract your partner (.87)	--
7. Tried to avoid him/her (.80)	--
10. Left home to get away from him/her (.88)	--
11. Ended (or tried to end) the relationship (.86)	--
12. Argued back verbally (.71)	--
13. Did nothing - just didn't do it (.78)	--

Note. Factor loadings are presented in parentheses

Table S5

Spearman's Rank Correlations for Scale and Composite Variables

	1	2	3	4	5	6	7	8	9	10	11	12	13	14	15	16
1. CIPR demand victimization	--	.71**	.81**	.76**	.86**	.67**	.74**	.94**	.82**	-.11*	.51**	.42**	.66**	.63**	.64**	.65**
2. CIPR threat victimization		--	.74**	.75**	.74**	.83**	.76**	.76**	.74**	-.19**	.57**	.48**	.76**	.73**	.76**	.72**
3. CIPR surveillance victimization			--	.70**	.79**	.71**	.79**	.84**	.77**	-.08	.50**	.39**	.70**	.67**	.67**	.67**
4. CIPR response to victimization				--	.77**	.71**	.74**	.75**	.73**	-.21**	.57**	.50**	.65**	.65**	.69**	.70**
5. CIPR demand perpetration					--	.76**	.82**	.85**	.94**	-.14**	.53**	.44**	.70**	.69**	.67**	.71**
6. CIPR threat perpetration						--	.80**	.73**	.78**	-.18**	.58**	.50**	.76**	.77**	.72**	.75**
7. CIPR surveillance perpetration							--	.78**	.83**	-.11*	.57**	.45**	.71**	.73**	.69**	.74**
8. CIPR total coercion victimization								--	.87**	-.12*	.54**	.42**	.71**	.68**	.68**	.67**
9. CIPR total coercion perpetration									--	-.13*	.54**	.42**	.70**	.70**	.67**	.71**
10. Social desirability total										--	-.28**	-.28**	-.13**	-.15**	-.19**	-.21**
11. PTSD total											--	.67**	.60**	.56**	.62**	.58**
12. Depression total												--	.47**	.44**	.51**	.47**
13. CCB physical violence victimization													--	.80**	.80**	.73**
14. CCB physical violence perpetration														--	.71**	.80**
15. CCB total coercion victimization															--	.78**
16. CCB total coercion perpetration																--

Note. *$p < 0.05$. **$p < 0.00$0
